# Challenges in the Early Diagnosis of Oral Cancer, Evidence Gaps and Strategies for Improvement: A Scoping Review of Systematic Reviews

**DOI:** 10.3390/cancers14194967

**Published:** 2022-10-10

**Authors:** Miguel Ángel González-Moles, Manuel Aguilar-Ruiz, Pablo Ramos-García

**Affiliations:** 1 Faculty of Dentistry, University of Granada, 18071 Granada, Spain; 2 Instituto de Investigación Biosanitaria ibs.GRANADA, 18012 Granada, Spain; 3 Faculty of Medicine, Complutense University of Madrid, 28040 Madrid, Spain

**Keywords:** oral cancer, diagnostic delay, early diagnosis, prognosis, scoping review, systematic review, meta-analysis

## Abstract

**Simple Summary:**

Oral cancer presents a worldwide incidence of 377,713 new cases and 177,757 deaths per year (GLOBOCAN, IARC, WHO). Oral squamous cell carcinoma (OSCC) accounts for approximately 90% of oral malignancies and has a 5-year mortality rate close to 50%. We aim to better understand, based on the evidence, and to discuss in depth, the reasons for the diagnostic delay of oral cancer by reviewing systematic reviews and meta-analyses. We also aim to, identify gaps in evidence, recommend future lines of research that should be implemented, and formulate strategies for improvement.

**Abstract:**

Oral cancer is a growing problem, accounting for 377,713 worldwide new cases per year, and 177,757 deaths annually and representing a 5-year mortality rate close to 50%, which is a considerable mortality that has not decreased substantially in the last 40 years. The main cause of this high mortality is related to the diagnosis of a high percentage of oral cancers in advanced stages (stages III and IV) in which treatment is complex, mutilating or disabling, and ineffective. The essential cause of a cancer diagnosis at a late stage is the delay in diagnosis, therefore, the achievement of the objective of improving the prognosis of oral cancer involves reducing the delay in its diagnosis. The reasons for the delay in the diagnosis of oral cancer are complex and involve several actors and circumstances—patients, health care providers, and health services. In this paper, we present the results of a scoping review of systematic reviews on the diagnostic delay in oral cancer with the aim to better understand, based on the evidence, and discuss in depth, the reasons for this fact, and to identify evidence gaps and formulate strategies for improvement.

## 1. Introduction

Oral cancer and oropharyngeal cancers are growing problems, respectively, accounting for 377,713 and 98,412 worldwide new cases per year, and 177,757 and 48,143 deaths annually (GLOBOCAN, IARC, WHO) [1]. Moreover, the considerable mortality of patients suffering from oral cancer has not decreased substantially in the last 40 years, with a 5-year mortality rate close to 50%. Almost the majority of oral cancers correspond to oral squamous cell carcinomas (OSCC), representing approximately 90% of mouth neoplasms [2,3]. Strikingly, approximately 50% of OSCCs are currently being diagnosed at an advanced tumour stage, with an increased tumour size (i.e., T3/T4), and 47% also harbours a N+ status [4,5]. Other data indicate that 30% of oral cancer patients delay seeking help for more than 3 months after they first notice signs and symptoms of the disease [6], to which must be added the time involved in the complex process of reaching a definitive diagnosis of oral cancer. Furthermore, up to 30% of the patients develop multiple tumours in 5–10 years [7,8], worsening the prognosis [9]. Considering the oral cavity is an anatomical region easy to be explored by dentists and physicians, the early diagnosis of oral cancer—with smaller tumour size and lymph nodes not affected by metastases—should be associated with a better survival [10,11,12].

The available evidence, although sometimes contradictory, seems to support that delay in the diagnosis of oral cancer strongly affects the prognosis of the disease. A recently published systematic review and meta-analysis [12] points to a significant association between diagnostic delay and the presence of tumours in advanced stages, deriving these data essentially from the studies of the highest methodological quality included in the aforementioned meta-analysis [10,13,14,15]; however, primary level studies on the subject that present results of case series and which, due to the characteristics of their design, provide a lower degree of evidence, sometimes present contradictory data. Therefore, in some tumour types and also in oral cancer, case series have shown a significant association between late diagnosis and the presence of advanced tumours [10,16,17,18,19,20]. Gomez et al. [10] reported that patients with late-diagnosed oral carcinomas were 30% more likely to be diagnosed with advanced stage tumours; other oral cancer series have not found this association [15,21,22,23,24]. These contradictory results are probably related to flaws in the methodology with which the studies were conducted (different concepts of diagnostic delay, retrospective designs without strategies to decrease the risk of patient recall bias, insufficient sample sizes, or heterogeneous intraoral locations introducing confounding factors into the analysis) [25,26,27]. In relation to survival, studies on the subject also routinely report that delayed diagnosis of oral cancer is associated with increased mortality [15,28,29]. Scott et al. [6] concluded that early detection of oral cancer is the best means of increasing survival and decreasing treatment-related disfigurement. It has also been reported that survival of patients treated within the first month of symptom onset is 86% at 5 years, whereas survival drops to 47% if the diagnosis is made within 7 months of symptom onset, and that after 12 months, the chances of survival are very poor [6]. It has been reported [15,28] that late diagnosis of oral cancer increased the mortality of patients in their series by 2.5 times and Seoane et al. [11] reported that early treatment of oral cancer improves the prognosis of patients and was associated with higher survival rates and better quality of life. 

The study of the influence of diagnostic delay on survival may be influenced by biases in the design of the analyses [30]. For example, some studies measure diagnostic delay as the time between the onset of symptoms or signs attributable to the tumour and the definitive histopathological diagnosis, without considering delays associated with late treatment initiation [31,32,33]. These may be particularly important because time to surgery, radiotherapy, and chemotherapy have been shown to be relevant prognostic factors for survival [34]. Likewise, some discordant or paradoxical results regarding the influence of diagnostic delay on survival may be due to the existence of confounding factors not considered in the analysis; for example, it could be that less aggressive tumours with a low growth rate, and therefore a priori better prognosis, behave in a less symptomatic or obvious way in their early stages and are diagnosed, for these reasons, later. This could explain the paradox that a greater diagnostic delay in this type of tumour could be associated with greater survival [12], and conversely, tumours with a higher growth rate and greater aggressiveness, will probably develop early symptoms that will facilitate their early diagnosis, although their prognosis will be clouded due to their biopathology [6]. 

From the above, it seems to be deduced that a primary objective to be achieved in the management of oral cancer, with clear repercussions on its prognosis, is its early diagnosis. Health authorities have undertaken screening programmes with different designs that aim to detect oral carcinomas at the earliest stages of their development when they are still asymptomatic, and academic authorities are increasingly encouraging health care providers to be aware of the clinical warning signs that should lead to the suspicion that oral cancer is developing. Despite this, evidence indicates that an unacceptable percentage of oral carcinomas are diagnosed late, which necessarily leads to high mortality. Understanding the reasons why a carcinoma developing in an area that is so accessible and frequently explored, such as the oral cavity, is diagnosed late is essential to overcome the challenge of achieving early diagnosis in most patients with oral cancer. This scoping review of systematic reviews aims to present the current state of evidence-based knowledge in this field, detect evidence gaps, and propose strategies to improve our ability to diagnose oral cancer early.

## 2. Materials and Methods

This scoping review adhered to the Preferred Reporting Items for Systematic reviews and Meta-Analyses extension for Scoping Reviews (PRISMA-ScR) [35].

### 2.1. Search Strategy

The databases MEDLINE (via PubMed), Embase, Cochrane Database of Systematic Reviews (aka Cochrane Library), and DARE, the Database of Abstracts of Reviews of Effects, were searched for systematic reviews published before July 2022, with no older date limit. The search strategy (Appendix A), considering PRESS initiative [36], was designed and conducted by combining thesaurus terms used by the databases (i.e., MeSH and EMTREE) with free terms, and built to maximize sensitivity. Keywords were combined jointly with an optimal search filter specifically designed by the Centre for Reviews and Dissemination-CRD for retrieving systematic reviews and meta-analyses [37,38]. An additional final screening was performed by handsearching the reference lists of retrieved included studies and using Google Scholar. All references were managed using Mendeley v.1.19.8 (Elsevier, Amsterdam, The Netherlands); duplicate references were eliminated.

### 2.2. Eligibility Criteria

We included systematic reviews, with or without meta-analysis, evaluating the diagnostic delay in the context of oral cancer. The term “oral cancer” was considered as a synonym for the term “oral squamous cell carcinoma”, having in mind that although these terms are not equivalent, almost the majority of oral carcinomas correspond to this histopathological lineage. A “systematic review” was defined as a review clearly formulating a research question and using systematic and explicit methods (minimally a search strategy and eligibility criteria) to identify, select, and critically appraise relevant research, and to collect and analyse data from the studies that were included in the review [39,40]. No restrictions were applied in relation to the publication language, publication date, characteristics of the secondary-level studies included in the systematic reviews (e.g., study design, geographical areas, sex and age of patients, and follow up periods, etc).

### 2.3. Study Selection Process

Eligibility criteria were independently applied by three authors (MAGM, MAR and PRG). Articles were selected in two phases: first screening titles and abstracts for articles apparently meeting inclusion criteria, and then reading the full text of the selected articles, excluding those that failed to meet the eligibility criteria. Any discrepancies were resolved by consensus. 

### 2.4. Data Extraction

Three authors (MAGM, MAR and PRG) extracted data from the selected articles, completing a data collection form in a standardized manner using Excel and Word (v.16/2018, Microsoft; Redmond, WA, USA). Data were gathered on the first author, publication year, journal, study population, sample size (i.e., number of studies), study design (i.e., systematic review with or without meta-analysis), and key results. These datasets were additionally cross-checked in several rounds, solving discrepancies by consensus.

### 2.5. Critical Analysis and Evidence Synthesis

A scoping review design seems pertinent to search for evidence-based results and potential evidence gaps [6,7]. The implications of diagnostic delay in oral cancer, investigated across systematic reviews, were explored to synthesize current evidence, search for potential evidence gaps, and guide future research. Key results were shown in descriptive tables, using a systematic methodological approach, and critically discussed in depth.

## 3. Results

### 3.1. Results of the Literature Search

**Figure 1:**
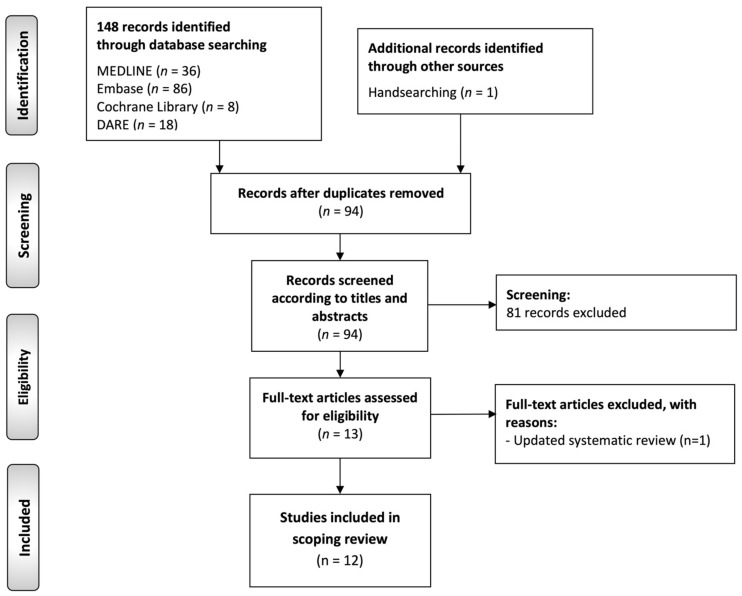
Flow diagram of the identification and selection process of the studies included in this scoping review of systematic reviews.

The flow diagram (Figure 1) depicts the results of the literature search, study identification, and selection process in this scoping review of systematic reviews. A total of 148 publications were retrieved: 86 from Embase, 36 from MEDLINE (through PubMed), 18 from DARE, 8 from Cochrane Library database of systematic reviews, and one by handsearching methods. After duplicate elimination, 94 records were considered potentially eligible and screened according to titles and abstracts, leaving a sample of 13 studies for full text evaluation. Finally, 12 studies meeting all eligibility criteria were included for critical analysis and evidence synthesis in our scoping review [10,11,12,30,41,42,43,44,45,46,47,48].

### 3.2. Study Characteristics 

Table 1 summarizes the characteristics of the 12 studies included in this scoping review. The first paper was published in 2006, and the most recent in 2022. All studies systematically reviewed primary-level studies, where the sample size varied(from 8 to 63 studies) and recruiting patients with oral cancer. According to the study design, all studies were secondary-level systematic reviews, and five of them performed meta-analysis (41.7%).

### 3.3. Critical Analysis and Evidence Synthesis 

Table 2 summarizes the objectives and key results derived from the published research on the implications of diagnostic delay in oral cancer, through secondary-level systematic reviews. The topics researched varied widely across the studies, and were categorized and ordered by relative frequency: the most investigated topic was the impact of diagnostic delay on oral cancer prognosis and clinico-pathological parameters (*n* = 5, 41.7%); followed by the existing knowledge of causes and factors influencing the diagnostic delay in oral cancer (*n* = 3, 25.0%); the time intervals and relative length of delayed diagnosis in oral cancer (*n* = 2, 16.7%); and the diagnostic accuracy of the diagnostic tests for the detection of oral cancer to provide more timely results (*n* = 2, 16.7%). The quality of evidence was formally assessed by four studies (33.3%, in all cases applying the Grading of Recommendations Assessment, Development and Evaluation [GRADE] system). As usually occurs in systematic reviews and meta-analyses of observational studies, the quality of the evidence was classified as low or very low for most of the outcomes critically assessed, which supports the need for future methodologically well-designed primary-level studies.

## 4. Discussion

### 4.1. Clinical Signs of Early Oral Carcinoma and Oral Lesions at Risk of Developing Cancer—Oral Potentially Malignant Disorders (OPMD)

**Figure 2:**
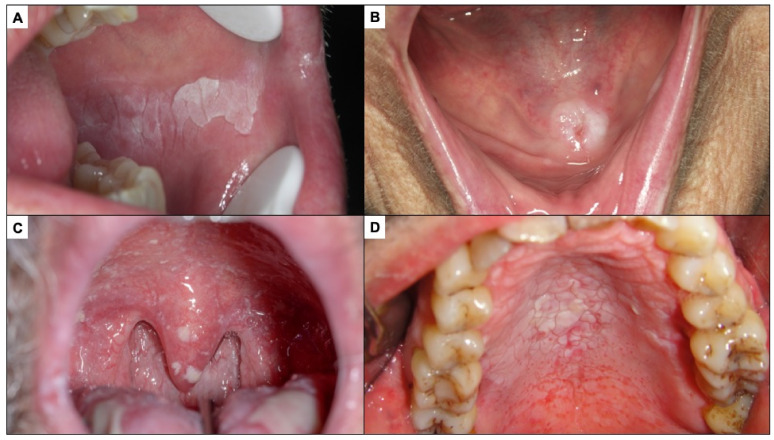
(**A**). Oral leukoplakia. (**B**). Frictional keratosis in the floor of mouth produced by a poorly fitted prosthesis. (**C**). Pseudomembranous candidiasis in an immune-compromised patient. (**D**). Nicotic stomatitis.

The first objective that should be achieved is that health care providers should precisely know the clinical signs and symptoms that lead to the suspicion that a patient has an early oral cancer or OPMD. In February 2020, the WHO Collaborating Centre for Oral Cancer through an expert group of 12 clinicians and researchers in the field of oral cancer and OPMD—to which MAGM, the main author of this paper, belongs—met in Glasgow with the aim of reaching and presenting a consensus on a revised classification of OPMDs, and recommended nomenclature and definitions for each disorder [49]. This working group considered that the oral lesions listed in Table 3 should currently be considered as OPMDs. The clinical manifestations of the most relevant OPMDs are presented below:

Oral leukoplakia is currently defined as a predominantly white plaque of questionable risk having excluded (other) known diseases or disorders that carry no increased risk for cancer [49] (Figure 2A). Therefore, the finding of a white lesion of the oral mucosa should make the clinician think of leukoplakia and, consequently, if the diagnosis is confirmed, the patient will be at risk of developing oral cancer in the future. The malignancy rate of oral leukoplakia ranges from 1 to 9% of cases, according to the most relevant studies published to date [49,50,51,52]. The diagnosis of leukoplakia is reached by exclusion of other white lesions of the oral mucosa that do not present any risk of progressing to cancer. In the experience of the authors of this paper, the oral white lesions that are most difficult to diagnose and most frequently raised in the differential diagnosis with oral leukoplakia are the following:

Frictional keratoses occur because of continuous rubbing on the oral mucosa, which induces hyperkeratosis (thickening of the corneal layer of the oral epithelium) that is clinically expressed as a white lesion. Frictional keratoses usually appear in areas of frequent rubbing—essentially lips, tongue, and the buccal mucosa (Figure 2B). The most common causes of chronic friction are teeth in poor condition and old, misaligned dentures. The clinician, when faced with a white lesion of the oral mucosa, should look for causes of friction and eliminate them. Frictional keratoses disappear within a week after elimination of the cause, which therefore serves as a diagnostic test and treatment. If the lesion does not disappear after elimination of the cause, an oral biopsy should be taken.

Pseudomembranous candidiasis is a fungal infection, usually *Candida albicans*, which manifests as white lumpy lesions resembling milk or yoghurt clots, which spread widely on the oral mucosa, especially along the hard and soft palate (Figure 2C). It is an opportunistic infection that appears in critically ill, immunocompromised patients, dehydrated or elderly patients, all circumstances that should be reflected in the patient’s clinical history or investigated by the clinician, helping to suspect the diagnosis. However, the most common clinical feature distinguishing pseudomembranous candidiasis from oral leukoplakia is that the former detaches when the lesion is scraped off with gauze; therefore, all white lesions of the oral mucosa should be scraped off as part of the diagnostic strategy (oral leukoplakias never detach on scraping).

Nicotinic stomatitis or nicotinic palatitis is characterised by the presence of a white lesion on the hard palate associated with red stippling secondary to inflammation of the excretory ducts of the minor salivary glands of the palate. In advanced stages, the lesion is also fissured, giving it a mosaic appearance (Figure 2D); it is caused by tobacco use, especially but not exclusively in pipe smokers, and usually disappears after cessation of smoking. The clinical features are sufficiently explicit for a proper diagnosis to be made. Although nicotinic palatitis is not associated with a risk of developing cancer of the palate, it is indicative of high tobacco use and thus the patient should be informed that there is a risk of developing multiple types of tumours and other tobacco-associated diseases.

**Figure 3:**
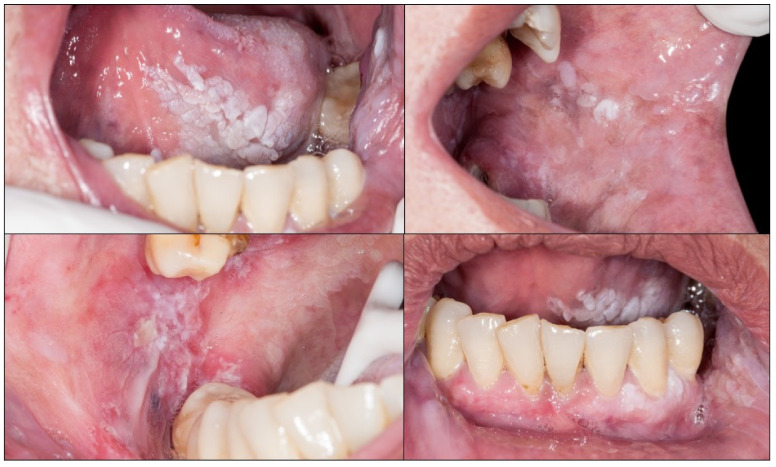
Proliferative verrucous leukoplakia. The presence of white multifocal lesions affecting the tongue, buccal mucosa, and gingivae should be noted.

**Figure 4:**
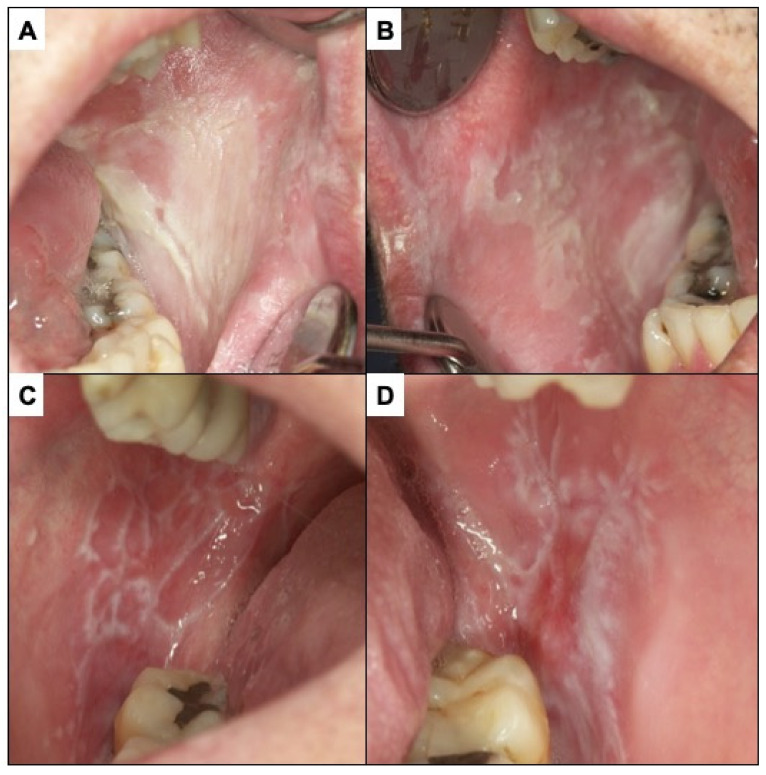
(**A**,**B**) White spongy nevus. It is a lesion that must be differentiated from PVL. It is a hereditary disease that appears in childhood without premalignant character. (**C**,**D**) Oral lichen planus. The presence of bilateral white reticular lesions characteristic of this disease should be noted.

Proliferative verrucous leukoplakia (PVL) is defined as a progressive, persistent, and irreversible disorder characterised by the presence of multiple leukoplakias that frequently become warty [49,53,54,55] (Figure 3). It is a lesion of unknown cause with a high risk of developing frequently multiple oral carcinomas (approximately 50% of patients with PVL develop cancer). This lesion does not detach on scraping thereby eliminating the possibility of confusion with pseudomembranous candidiasis. White spongeous nevus (WSN) may have a similar appearance to PVL, although WSN is an autosomal dominant inherited disease that affects more than one member of the same family (necessarily one parent), appears in childhood (which is never the case in PVL) and is not a disease at risk for progression to cancer (Figure 4A,B).

Oral lichen planus (OLP) is defined as a chronic inflammatory disorder of unknown aetiology with characteristic relapses and remissions, displaying white reticular lesions, accompanied or not by atrophic, erosive, and ulcerative and/or plaque type areas. Lesions are frequently bilaterally symmetrical. Desquamative gingivitis may be a feature (Figure 4C,D). The reticular appearance of the lesion is highly suggestive and does not usually pose major diagnostic problems if the lesion is known. The risk of malignancy of OLP is slightly more than 1% of cases. However, atrophic, and erosive lesions on the tongue, especially if the patient is a smoker, present a higher risk [49,56,57,58,59,60,61]. A major challenge in the clinical follow-up of patients with OLP regarding the early diagnosis of malignant transformation of the disease relates to the difficulty in differentiating the atrophic–erythematous areas that frequently appear in OLP from those areas that represent erythroplasias, the expression of incipient carcinomas. Although the physician’s experience is essential here, some facts may be indicative of early carcinoma. These include the good delimitation that erythroplasias usually present—as compared to the more diffuse aspect of non-malignant inflammatory erythematous areas—and the roughness that incipient carcinomas usually present. Nevertheless, clinicians should be aware of these difficulties and be especially alert to the possibility that an atrophic erythematous area in a OLP may represent an area of incipient malignancy.

**Figure 5:**
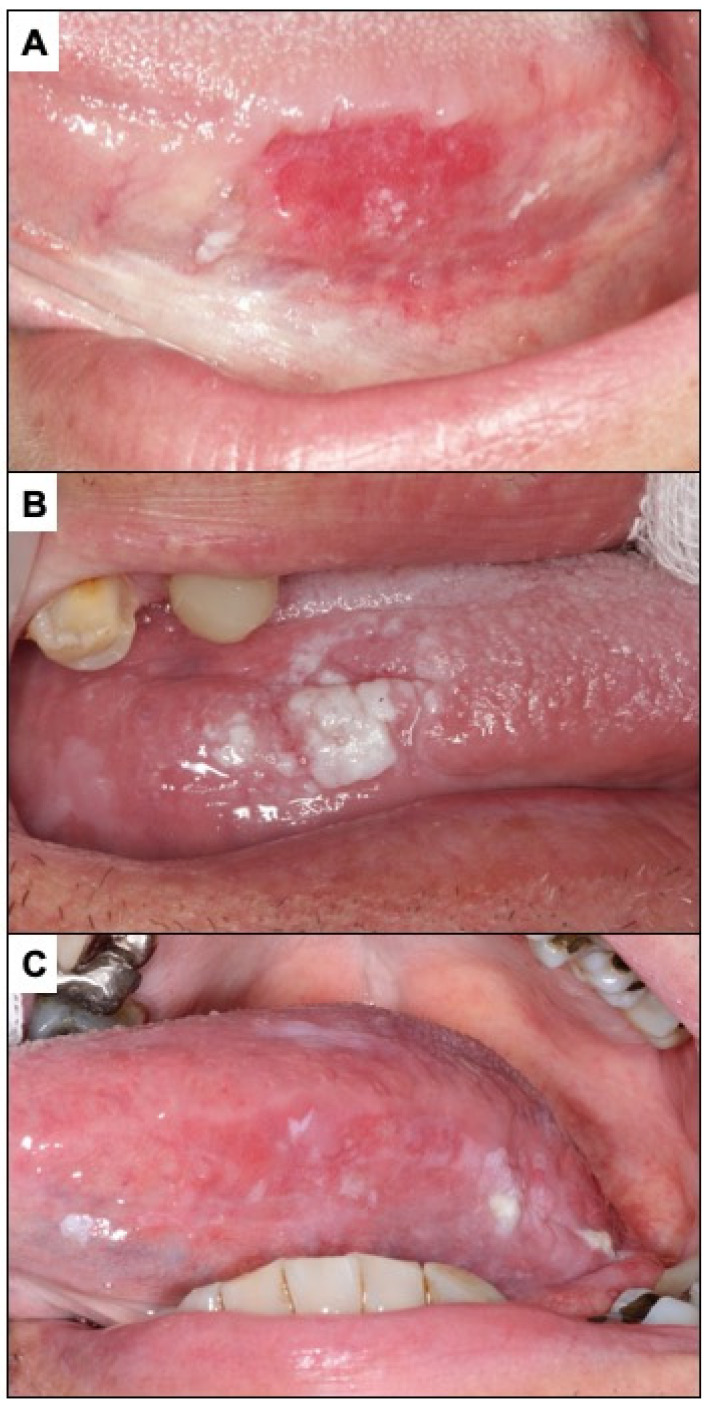
(**A**). Erythroplasia of the lateral border of the tongue. (**B**). Oral carcinoma with leukoplakia-like appearance on the lateral border of the tongue. (**C**). Oral carcinoma with a red and white appearance.

Erythroplasia is defined as a predominantly fiery red patch that cannot be characterised clinically or pathologically as any other definable disease [49] (Figure 5A). More than 70% of oral erythroplasia are established carcinomas, carcinomas in situ or have severe dysplasia. The differential diagnosis of oral erythroplasia is complex because numerous inflammatory and traumatic lesions of the oral mucosa can manifest with an erythematous appearance. The presence of well-defined limits in a lesion involving a localised area of the oral mucosa is suggestive of erythroplasia. 

Our research group, in two systematic reviews and meta-analyses [53,60], has shown that carcinomas that develop on OLP and PVL behave with a better prognosis compared to those that do not occur on OPMDs. The reason for this more favourable behaviour is unknown, although some facts suggest that this may be related to the biopathology of the tumour itself and not to the early diagnosis that hypothetically should be made in some oral carcinomas that arise in these lesions, because of the desired close follow-up of these lesions, which on the other hand is not usual.

**Figure 6:**
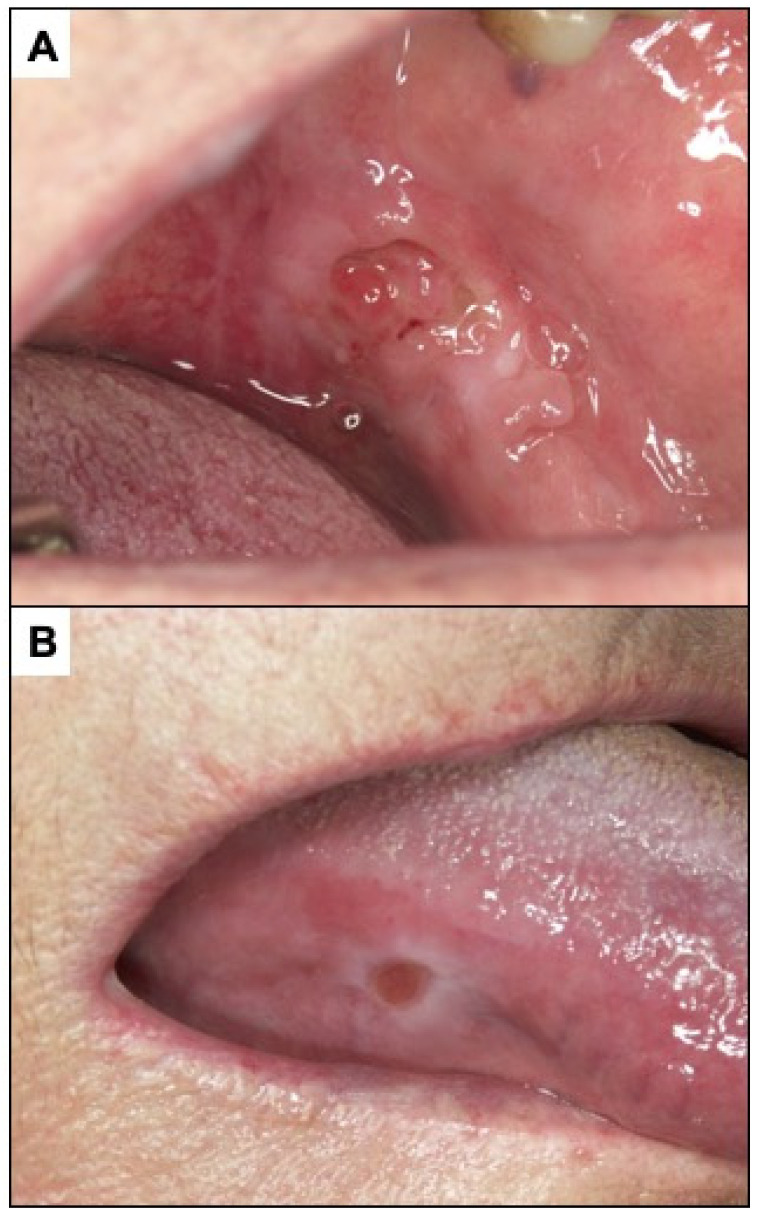
(**A**). Ulcerated incipient oral carcinoma. (**B**). Benign traumatic ulcer. The characteristics of this ulcer should be noted. Clean and homogeneous bottom, well-defined border, white peri-ulcerous halo.

**Figure 7:**
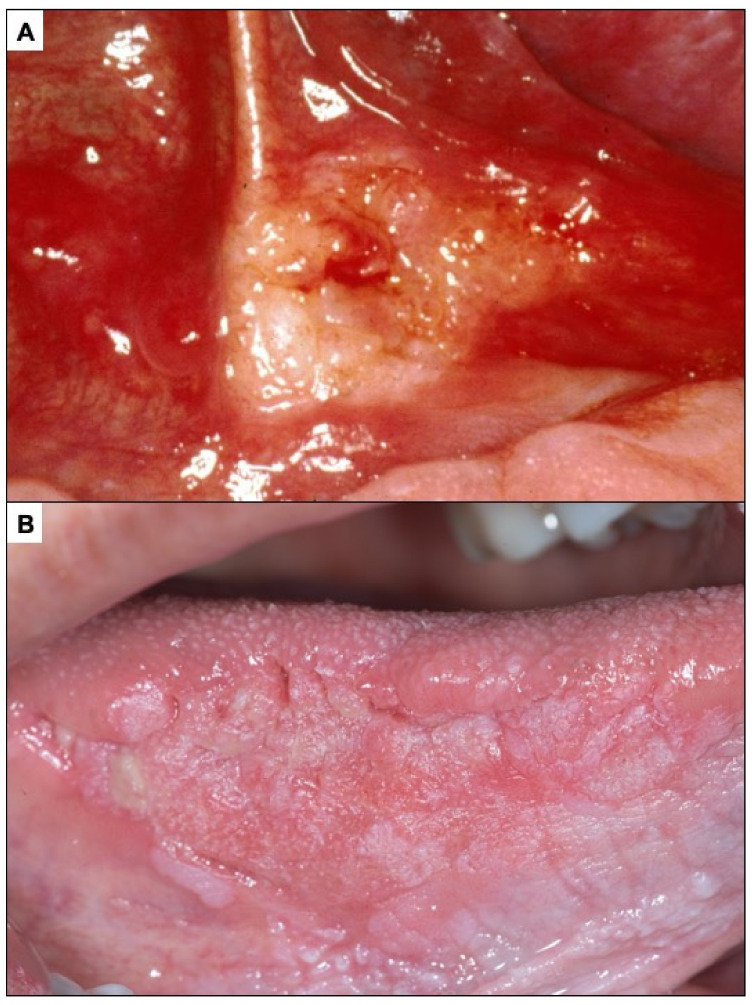
(**A**). Incipient oral carcinoma appearing as a raised lesion. (**B**). Oral carcinoma with granular aspect.

Oral carcinomas in their early stages present in more than 60% of cases as erythroplastic areas (Figure 5A). About 12% of oral carcinomas present initially as white areas similar to leukoplakia (Figure 5B) and may also appear as a mixture of red and white lesions (Figure 5C). The presence of chronic ulcerations (more than one month of evolution), not justified by chronic trauma, should suggest cancer (Figure 6A), especially if they are hard to the palpation and present irregular raised edges and dirty and distorted bottoms. Benign traumatic ulcerative lesions are characterised by well-defined margins, homogeneous ulcerative surfaces with the presence of a peri-ulcerative white halos (Figure 6B). The underlying traumatic cause is usually found and must be eliminated. Patients in such a case should be reviewed in two weeks to confirm that the lesion has disappeared; if not, a biopsy is necessary. It is possible that an incipient oral carcinoma may also present as a raised lesion (Figure 7A) or a granular lesion (Figure 7B). As mentioned above, malignant lesions are usually hard or rough to the palpation.

### 4.2. What Is Early Oral Carcinoma

**Figure 8:**
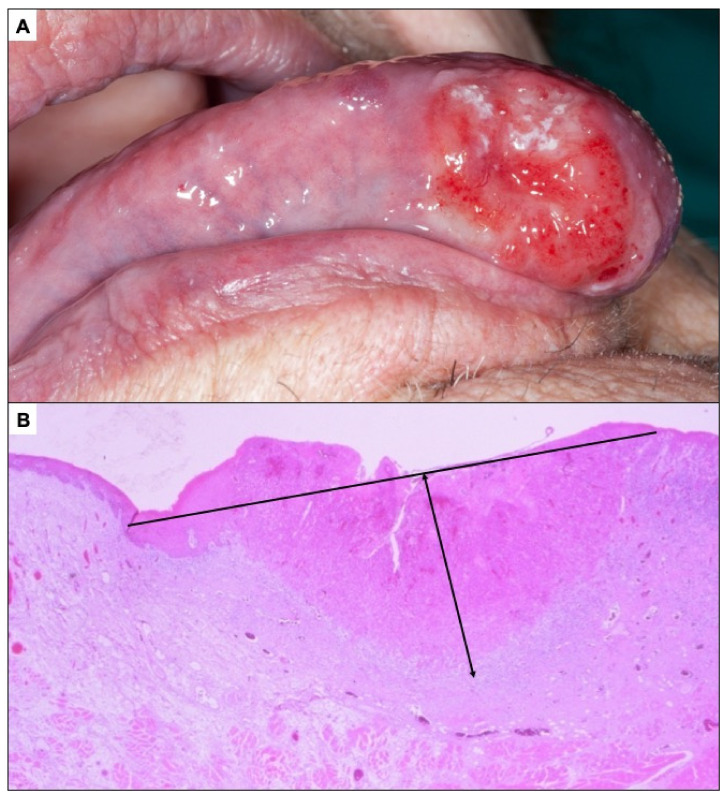
(**A**). Oral carcinoma less than 2 cm in greatest diameter whose classification as an incipient carcinoma is questionable. (**B**). Oral carcinoma less than 5 mm depth of invasion.

Common sense dictates that an incipient oral carcinoma should manifest as a small lesion. However, in a disease that progressively grows, except in extreme cases, it is difficult to establish precise demarcation between small and larger carcinomas. On the other hand, a small carcinoma does not necessarily correspond strictly to a carcinoma that is starting to develop, and thus, some aspects of the particular biopathology of each tumour—for example, a low proliferation rate—may condition slow-growing tumours to manifest themselves as small tumours for a long time; and conversely, very proliferative, fast-growing tumours may present as large tumours shortly after their establishment. Nevertheless, in general it seems prudent to accept that a small tumour is likely to be a tumour that has only recently developed, and conversely, we could assume that large tumours have a longer evolution time. The T—tumour size—parameter in the AJCC classification (Tclinical-Tc-: maximum diameter of the tumour before being removed, or pathological T-Tp-: maximum tumour diameter in the surgical specimen) is the most commonly used parameter to determine whether a tumour is small or not, and thus, T1 tumours are considered small tumours because it has been shown that their prognosis is significantly better than that of oral carcinomas with T stages greater than 1 [62]. According to the latest AJCC TNM classification [63,64], a T1 oral carcinoma should measure less than 2 cm in greatest diameter, and thus, on visual inspection of the oral cavity a small oral carcinoma should measure less than 2 cm. Figure 8A presents an oral carcinoma of less than 2 cm in greatest diameter, which would, therefore, theoretically have been diagnosed early; however, this image would not satisfy any clinician with experience in oral cancer, who could hardly accept that the diagnosis of this carcinoma has been made early. This case, and many others like it, illustrates the difficulty in conceptualising what should really be considered as early carcinoma. The better prognosis of small oral carcinomas is determined by their lower probability of having metastasised to tissues far from the oral cavity, essentially in the lymph nodes of the neck (N+ in the AJCC system), at the time of diagnosis, a fact that is widely recognised as one of the most drastically affecting the prognosis of patients [62]. For an oral carcinoma to be able to metastasise to the lymph nodes of the neck, it must reach the lymphatic vessels found in the chorion of the oral mucosa, i.e., it must invade deeply into the oral mucosa. The depth of tumour invasion is now considered to be one of the parameters that best predicts the prognosis of patients with oral cancer. Our research group reported years ago that oral carcinomas with invasion depths greater than 3 mm had significantly lower survival rates [62] and currently, the AJCC has included the tumour depth of invasion among the conditions of the T parameter, so that tumours with >5 mm depth of invasion would be considered T2 [63,64]. Therefore, we could accept that the diagnosis of an oral carcinoma has been made early if it is less than 5 mm in deep (Figure 8B).

### 4.3. What Is Delayed Diagnosis in Oral Cancer and How to Investigate It

Logic dictates linking the concept of diagnostic delay to the parameter time, which would refer to the passage of an excessive amount of time between the appearance of the first sign of an oral carcinoma and its definitive diagnosis-diagnostic time [10,12], and this has been considered in this way by many authors since the 1970s [6,17,23,24,32,65,66]; however, the conceptualisation of diagnostic delay as a time delay has as its first drawback the lack of consensus on from which point (time point) a diagnosis of oral cancer should be considered delayed [10,12]. Some authors for comparative and statistical purposes, have considered the delay as the mean or median time to definitive diagnosis [16,21,67]; others have defined a delayed diagnosis as that exceeding an arbitrarily chosen time (e.g., >30 days) [16,25,68]; in other cases authors have divided the diagnostic delay into intervals (<1 month, 1–3 months, >3 months) [69] or have considered the diagnostic time as a continuous variable without setting a specific cut-off point to differentiate cases where the diagnosis has been delayed [23,70]. In this sense, logic also dictates that we should only consider a diagnosis of oral cancer to have been late if this has had determinative consequences for life or quality of life for the patient, i.e., if because of a late diagnosis the patient is very likely to die or to be subjected to a mutilating or disabling treatment. Because what determines the prognosis of an oral carcinoma is essentially its size at the time of diagnosis—especially its depth of invasion or tumour thickness—and the lymph node involvement, which is also strongly, but not exclusively, dependent on size, it seems that the diagnosis of a large oral carcinoma is a more accurate indicator of late diagnosis than the time elapsed from when the patient perceives the first symptoms of cancer to its definitive diagnosis. Although large tumour size is obviously very often associated with a longer time of tumour evolution, diagnosis does not depend exclusively on time but also on some characteristics of tumour biopathology, i.e., aggressiveness, growth rate, degree of differentiation. For these reasons, the authors of this paper advise considering tumour size at the time of diagnosis, essentially referring to the depth of invasion, as a parameter to determine that the diagnosis of an oral carcinoma has been delayed. In other words, a tumour would have been diagnosed late if it is more than 2 cm in diameter at diagnosis, but especially, more than 5 mm in tumour thickness.

The challenge of improving cancer prognosis through early diagnosis requires a deeper understanding of the different actors, periods and conditioning factors involved in the diagnostic process. In 2012, a group of experts published the Aarhus declaration [71], a consensus document whose main objective was to propose and discuss a standardised set of definitions and requirements that should be met by research designs on early cancer diagnosis. The Aarhus statement consists of a description of events that take place during the pathway to cancer diagnosis [71]. It sets out five events—detection of body changes, perceived reasons for discussing symptoms with a health professional, first consultation with a health professional, diagnosis, and initiation of treatment. These events in turn define four time periods: the patient’s assessment of the clinical events he/she is experiencing and their consideration as abnormal. Help-seeking, which includes the period between the patient’s inference that he/she is ill until seeking professional help. The diagnostic interval, which covers the period from the first consultation with a health professional to the definitive pathological diagnosis [71] and includes a first investigation by a health professional, usually in primary care (family doctor, primary care or private dentist), the first referral to a specialist (maxillofacial surgeon, specialist in oral medicine and pathology, oncologist), the first visit by a specialist, and the establishment of a definitive histopathological diagnosis. Finally, the pre-treatment interval includes the planning of the most appropriate treatment for a patient with oral cancer depending on the stage of their disease and ends when treatment is initiated; surprisingly, the pre-treatment interval is not included in many studies on delayed diagnosis [30,71,72]. The Aarhus statement advises that research on delayed diagnosis of cancer should include four strategic dates: date of first symptoms/signs, date of first consultation with a health professional—usually in primary care, the date of referral—when a primary care professional refers the patient to a specialist in cancer diagnosis or management, and date of definitive diagnosis-histopathological diagnosis [6,12,71]. As we will see, all the above-mentioned intervals can be subject to delay in the diagnosis of oral cancer.

The Aarhus statement also considers several contributing factors that can lead to delayed diagnosis. These include factors dependent of the tumour itself—growth rate, tumour location, degree of differentiation, among others, for example, tumour location in the posterior third of the lateral margin of the tongue, where tumours grow silently to large sizes unnoticed by the patient, is considered to be a conditioning factor for diagnostic delay [15,67,73,74,75]. Contributing factors also include those dependent on the patient [76]; these essentially refer to the social and cultural circumstances which, as we shall see, will lead to a misinterpretation of the initial signs of oral cancer and consequently lengthen the assessment interval.

### 4.4. Reasons for Delayed Diagnosis of Oral Cancer

The reasons for delayed diagnosis of oral cancer are many and varied and understanding them is essential to implement strategies to overcome the challenge of achieving early diagnosis in most oral cavity carcinomas. The reasons for delay in diagnosis can affect each of the intervals that make up the overall time to diagnosis of oral cancer. 

**Figure 9:**
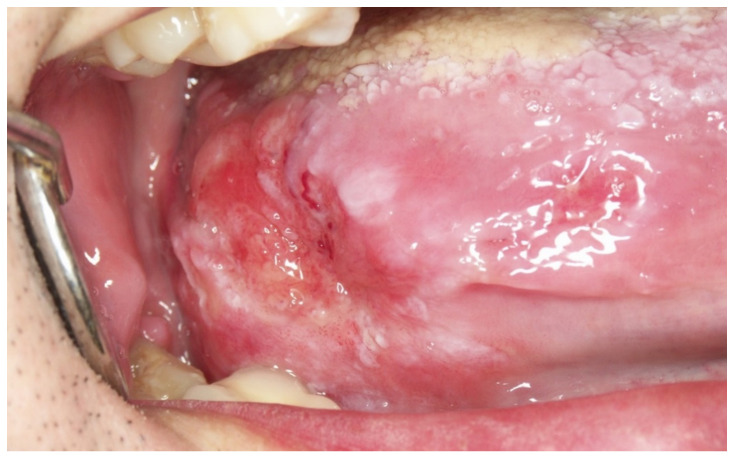
Advanced tumour growing silently on the lateral border of the tongue.

One group of major causes of diagnostic delay concerns patients. The oral cancer patient is directly responsible, consciously, or unconsciously, for the length of the so-called assessment interval (time between perception of the first symptom and seeking help). Some studies have pointed out that the delay in diagnosis attributed to the patient takes on the greatest significance within the overall delay in the diagnosis of oral cancer with an average of 104.9 days of delay attributable to the patient [29]. For a patient with oral cancer actively seek medical help, they must perceive the symptoms as abnormal, and this is made more difficult by several circumstances. First, oral carcinomas are often asymptomatic in the early stages of their development or appear in areas (e.g., deep lateral margin of the tongue) where lesions are not visible (Figure 9).

**Figure 10:**
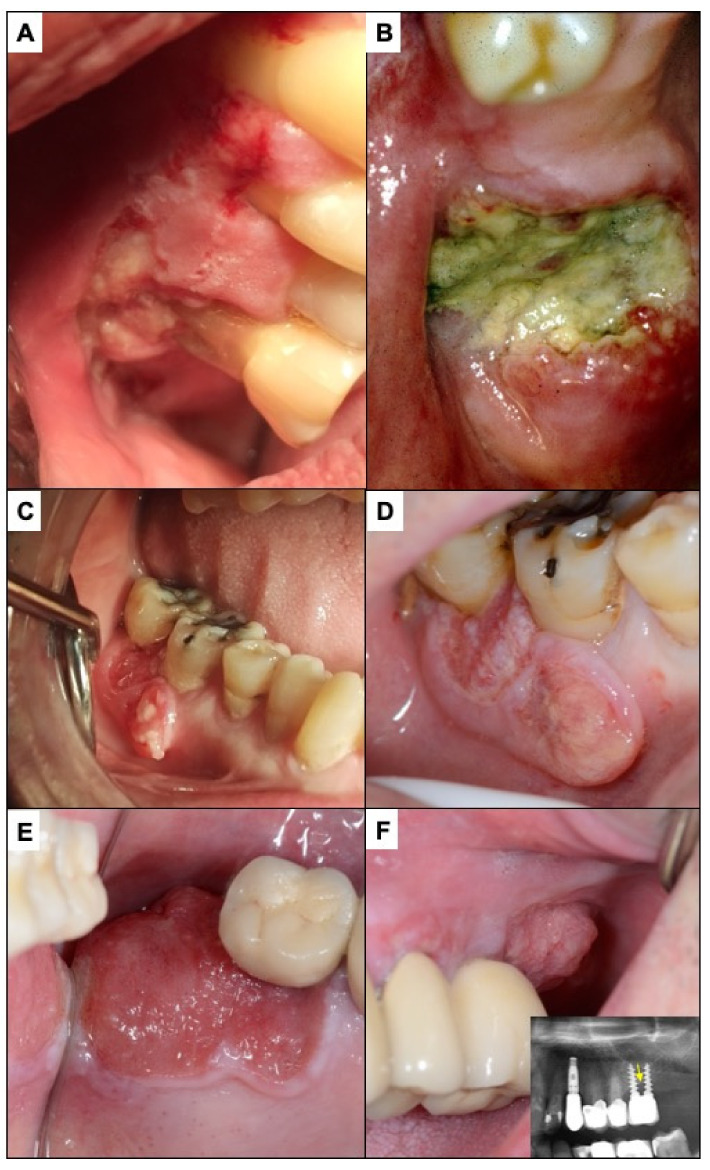
(**A**) The lesion presented by this patient was interpreted by the dentist as being consistent with periodontal disease; the biopsy showed that it was an oral carcinoma. (**B**) In the patient in the image, the neoplastic lesion located around the molar was interpreted as secondary to a periodontal process (periodontal disease). The dentist decided to extract the tooth and approximately 2 months later referred the patient for consultation due to a lack of healing of the extraction. (**C**) The carcinoma surrounding the lower right first and second molars in this patient was attributed by a private general practitioner general dentist to an infectious process affecting these molars and consequently the patient was treated with an antibiotic. Some time later the progressive growth of this neoplastic lesion was verified, where probably a worsening of the prognosis had already occurred (**D**). (**E**) corresponds to incipient carcinoma around an implant-supported upper molar. (**F**) shows how a mass with an evident neoplastic aspect is growing on the posterior part of the molar. This tumor lesion whose diagnosis has been delayed infiltrated the maxillary bone supporting the implants (radiographic image), which places this tumor in a T4 stage with a poor prognosis.

Patients with oral cancer often attribute their initial symptoms to banal reasons, such as traumatic ulcers due to tooth or prosthesis rubbing, accidental chewing trauma, non-specific inflammation, common aphthous ulcers, etc. Some studies have reported that the assessment interval is significantly lengthened for these reasons [17,77]. The social and cultural circumstances of the patient also seem to condition a misinterpretation of the initial symptoms of cancer. Therefore, patients from low social strata living in poverty, elderly patients living alone, institutionalised patients, homeless people, illegal immigrants, cognitively impaired patients, black, African-American or Hispanic patients (in the USA), may have difficulties in interpreting, communicating their symptoms, or even in establishing the necessary help-seeking processes [6,76,78,79,80,81]. In the authors’ experience, carcinomas that grow around teeth affecting the periodontal space cause symptoms—tooth mobility—that are often attributed by patients and even by health professionals to dental pathologies (Figure 10). 

Furthermore, relatively often—and in the authors’ own experience—especially in depressed social and cultural strata, patients may develop the idea that surgical intervention on the lesion could accelerate—trigger—the growth of the lesion and lead to their death. On the other hand, patients who begin to consider the idea that their symptoms may be the consequence of cancer development often experience a fear of receiving the news and of facing the likelihood of death or the unpleasant and painful events that will be involved in treating their disease, and this may lead to a delay in seeking help. The delay in diagnosis may also depend on difficulties in accessibility to health care, which will affect the interval between seeking help. Difficulties related to accessibility may depend on the circumstances of the patient—already discussed—or of the healthcare system. Accessibility is defined as the ability to obtain the required services based on health needs [82,83]. Accessibility may be limited by the marked lack of health care providers in some countries of the world (dentists, family doctors) [84] or by geographic constraints—isolation of populations in countries with large land areas, lack of means of transport in poor countries, complex local topography—which may also limit accessibility to health care [85].

Delays in diagnosis are sometimes due to health system or health provider-related causes, which contribute to an increase in the diagnostic interval. The first condition that a health care provider (essentially family physicians and dentists) must fulfil is to know the symptoms and signs of early oral carcinoma. Some authors have reported the ignorance of primary care providers about the symptoms of oral cancer [22,86] as very worrying. In the experience of the authors of this paper, many delays in the diagnosis of oral cancer attributable to health care providers are also due to an inexplicable lack of alertness and disregard for the possibility that a patient with an oral mucosal lesion might have carcinoma, which has also been reported by other authors [87]—you cannot diagnose what you do not think about. Sometimes, health care providers in primary care feel inhibited in the act of referring patients with suspicious lesions for fear of making a mistake and risking their prestige by being judged by other colleagues. On the other hand, the saturation of work to which physicians and dentists are subjected in primary care in many countries of the world leads to a relaxation of the basic principles of the routine examination to which all patients should be subjected [88]. It is also possible that a health care provider in primary care may decide to institute a treatment—chlorhexidine or other mouthwashes, hyaluronic acid, antifungal drugs, or even topical corticosteroids—not indicated and without enough justification, or even to make a new appointment sometime later (in some cases, months later) without performing any intervention, in the hope that the lesion will resolve spontaneously. Finally, it has been reported that the existence of comorbidities in patients with oral cancer makes clinicians prone to pay more attention to the symptoms of pre-existing diseases and not to new symptoms [24]. The diagnosis of oral cancer is complete when a histopathology report is given, which necessarily requires taking a biopsy of the lesion. Most often, the biopsy is taken in specialty care by a maxillofacial surgeon or a specialist in oral medicine and pathology. However, the oral mucosal biopsy procedure is very simple and could be performed in primary care by dentists or family doctors. This would considerably shorten the diagnostic interval as it would avoid referral to a specialist and would facilitate the referral of a case already diagnosed and ready to start the treatment process directly [6,33,82,85,89,90,91,92,93]. However, very few general dentists perform oral biopsies (between 7% and 32% as published for different countries in the world) [94,95,96,97,98,99,100]. Specific training in this field would solve this problem. Another possible way to reduce the time elapsed between a private dentist finding a possible incipient case of cancer in his clinic and the definitive diagnosis by a specialist, could be the direct referral of the patient to the specialist avoiding the previous step by a family physician. It is evident that only the private dentist should follow this procedure when he is convinced of the evident neoplastic aspect of an oral lesion. Otherwise, this would refer many false positive cases and would unnecessarily saturate specialized diagnostic services.

Finally, the delay in treatment planning, secondary to the saturation of health services, also necessarily worsens the prognosis of patients.

### 4.5. Improvement Strategies in the Early Diagnosis of Oral Cancer

In many countries of the world, the most common strategy for early diagnosis of oral cancer is active case finding; this is the term used to refer to patients presenting with abnormal signs and symptoms, who come to the clinic for this reason, and who should undergo a diagnostic test (biopsy and histopathological study) [101]. At this point, the improvement of the results in the early diagnosis of oral cancer depends on the training of the dentist, the family physician or other professionals who could be consulted by the patients. However, the personal commitment of health care providers to the proper development of their functions, their ethical level, their ability to overcome daily problems and not to lose heart in the face of the daily problems of medical care and to consider the patient as the crucial element of their activity, to whom they owe everything, are, in the opinion of the authors, obligatory circumstances in the improvement of the results of early diagnosis.

The high mortality rates associated with late diagnosis of oral cavity cancer have prompted some countries to institute screening programs in an attempt to identify patients with asymptomatic early-stage lesions by means of screening tests. Screening programs are organized, essential public health programs that have, when indicated, great potential to improve population health outcomes; when effectively organized, they can prevent disease, reduce disability and mortality. These programs apply screening tests that are not pretended to be diagnostic but to find patients with abnormal findings and to accelerate the referral and the application of specific diagnostic procedures by the specialist who will perform a re-examination and, if deemed necessary, a biopsy and a definitive histopathological diagnosis [101]. The conventional screening test in most screening programs is the systematic visual inspection under a brilliant light source and the palpation of the oral cavity and neck, which aims to find abnormalities that generate the suspicion of oral cancer or enlargements in the neck that could correspond to lymph node metastases [101]. 

There are different types of screening programs: opportunistic screening, population-based screening, workplace screening, and self-exams [101]. 

Opportunistic screening is a non-systematic activity that is usually performed within the health services taking advantage of a consultation for another medical reason. In the case of oral cancer, opportunistic screening is essentially performed by dentists and family practitioners on patients who come for consultations for other reasons. This type of screening can also be performed by other specialists (dermatologists, otolaryngologists, maxillofacial surgeons) who very often have access to oral cavity examination. Visual examination of the oral cavity is the test commonly used in opportunistic and population-based oral cancer screening programs. There are not many studies that have evaluated the efficacy of opportunistic screening in the early diagnosis of oral cancer, although in general terms, the authors point out that it is a feasible procedure [102], with the ability to detect oral cancer [103], although this seems to be greater in the detection of OPMDs versus oral cancer screening [104]; one advantage is that it does not take up excessive time in addition to that required by the previously scheduled patient visit and could be cost-effective [105]. Some authors even advise that this opportunistic screening should be repeated on more than one occasion [106]. On the other hand, it has been reported [107,108] that, although there is insufficient evidence to support or refute the efficacy of opportunistic screening in oral cancer [109], dentists and family physicians should continue to apply it as part of their routine activity. Finally, the data published to date seem to indicate that opportunistic oral cancer screening by visual examination is most effective in individuals with risk factors (i.e., heavy smokers and/or drinkers, or betel users) [110,111,112,113,114,115,116]. During the last few years some adjuvant methods and techniques have been presented and even commercialized as a complement to the visual examination of the oral cavity [117,118]. These types of aids are based on technologies that apply light of different wavelengths, sometimes with prior application of a photosensitizing drug (Vizilite plus, Microlux DL, Orascopiptuc DK, KED Dental, VELscope, Identafi, Illum sacan) [101,119,120], cytological techniques (Oral CDX) [117] even using liquid-based cytology, saliva protein detection techniques that have been shown in some studies to be increased in oral cancer patients (Il-6, Il-8, SCC-Ag2, Calcinin, 70 Kd heat shock protein, annexin I, cathepsin G, peroxiredoxin II, Thioredoxin, etc) [101,121], miRNA study techniques [122] and DNA quantification techniques [123]. The available evidence indicates that none of the adjunctive tests investigated can be recommended as a substitute for the currently used standard of surgical biopsy and histological evaluation [44].

A population screening program is actively offered to the whole target population, in a systematic way and within a regulated framework of public health policy, protocolized and with an adequate continuous evaluation of quality and results; it can be established by means of home visits or by invitation to attend the screening events. In relation to the application of this type of program for the early detection of oral cancer, the few studies on the subject conclude that there is insufficient evidence to defend the implementation of these programs, similar to what occurs in other types of tumours (breast cancer, cervical cancer or colon cancer) [107,108].

The main problems detected in oral cancer screening programs are detailed below:

- The occurrence of a high number of false-positive cases referred for confirmatory diagnosis has been noted. This is an aspect that considerably undermines the development of the programs as it consumes health resources unnecessarily, both in terms of time invested by specialists, as well as economic, and generates unnecessary stress in patients. This aspect is difficult to reverse and only the training of the examiners would reduce the number of false-positive cases referred.
- Population-based screening programs have proven to be very cost inefficient in countries where there is a low incidence of oral cancer.
- The poor compliance of patients selected as cases in a screening program when they are referred to a specialist for definitive diagnosis is very remarkable. This is a major problem that necessarily diminishes the effectiveness of screening programs and, in our opinion, could only be solved by improving the communication and information provided to selected patients by screeners.
- Variability in the training levels of examiners also affects the effectiveness of screening programs. This is because a program that aims to screen large populations should be supported by many examiners, which will necessarily make their level of training heterogeneous. Moreover, the resolution of this problem is hampered by the subtlety of the initial clinical manifestations of oral cancer. Only more in-depth training programs for examiners can improve this aspect.
- Lack of knowledge on the part of the examiners of the most common toxic habits in the population to be examined will prevent the selection of patients at higher risk, who are otherwise the main target of oral cancer screening programs.
- Screening programs are not usually designed in the form of randomized controlled trials, which are those that allow cases to be randomly assigned to groups and results to be effectively compared.
- Finally, the low level of resources in the countries targeted by screening programs is an aspect that greatly hinders their implementation. It should be taken into consideration that oral cancer is often more prevalent in poor societies.

### 4.6. Results of the Main Oral Cancer Screening Programs in the World

Three screening programs aimed at the early detection of oral cancer have been carried out that in our opinion deserve to be discussed in this paper:

The Cuban program was developed between 1982 and 1997, with the aim of improving the stage at which oral carcinomas are diagnosed in that country [124,125]. This program has an opportunistic design in the dental clinic and covered a population of 10 million patients, of whom 0.3% presented some sign of suspected oral cancer. A major problem encountered in the analysis of this program was that a small percentage of the cases detected in the program (only 29%) were reviewed by a specialist. Evaluation of the results of this program indicates that the number of cases diagnosed in stage I increased and the number of cases diagnosed in advanced stages decreased; it is also interesting to note that 16% of the 4412 oral carcinomas diagnosed in Cuba during the period 1982–1990 were diagnosed thanks to this program; however, no reduction in oral cancer incidence or mortality could be identified since the introduction of the Cuban program, although reports on the program ceased in 1997.

The Kerala (India) study, a randomized controlled trial, conducted between 1994 and 2009, aimed to reduce oral cancer mortality in the population examined. Eligibility criteria were all healthy subjects aged 35 years or older, with no personal history of oral cancer. Informed consent was signed by each participant. The intervention arm consisted of 96,517 participants and the control group consisted of 95,356 participants. During this period, four rounds of screening were conducted in which 91% of the target population was screened at least once. The essential results of this program point to a benefit of the screening program in high-risk subjects (smokers, drinkers, or betel users), as after the third round of screening a significant 34% reduction in mortality was detected in high-risk subjects and after the fourth round a sustained reduction in mortality of 81% and a decrease in oral cancer incidence of 38% was proven in the screened population compared to the control group [126,127]. The significant findings of this randomized controlled trial are widely recognized [101].

Finally, a program was conducted in Taiwan between 2004 and 2009 that evaluated the impact on patient survival [128]. More than 2 million adult smokers and/or betel consumers in Taiwan were invited to a dental check-up by the dentist, of whom 51% formalized their participation; of the participants, 4110 had oral cancer that was discovered at the first screening test. The essential results of this program demonstrate an increase in the percentage of cases diagnosed at early stages (46.5% vs. 39.6% in stage I and II in the comparison between cases that attended vs. cases that did not attend—control group), a 26% reduction in mortality in the screened group and a reduction in the incidence of oral cancer—133.4 cases/100,000 inhabitants in the study group versus 190.9 cases/100,000 inhabitants in the control group). Taiwan is the only country in the world that has implemented a sustained oral cancer screening program, and it should be noted that it currently offers screening to high-risk patients (betel chewers or former betel chewers and smokers) [101].

From the above it seems to follow that screening programs can be effective when targeted to populations at high risk of developing oral cancer [107]; thus, Downer et al. [129] has reported that targeted screening of high-risk population could save two to three times more lives than non-targeted screening, and in the USA [130,131] it has been reported that these programs are more cost-effective if targeted to male smokers and drinkers over 40 years of age.

### 4.7. Problems of Screening Programs

Among the essential problems that have been identified in screening programs [104] are the high percentage of false positive referrals, that is, cases that have been referred to the specialist under the suspicion of cancer that finally presented a benign lesion. The main drawback of this situation is that it unnecessarily consumes time and resources in specialized care and generates unjustified stress in the patient. This problem is accentuated by the fact that between 5% and 15% of the general population may have an oral mucosal lesion that in most cases will be benign and that visual inspection alone is not sufficient to adequately categorize it [117]. The low incidence of oral cancer in certain populations is also a problem for screening programs because it decreases their effectiveness. It has already been discussed that in these situations, screening performance improves if programs are targeted to at-risk populations, whereas community-based screening programs would be more cost-effective in high oral cancer incidence populations [104]. Areas of the world where community-based screening programs are more cost-effective because of higher oral cancer incidence are northern France, India, some areas of central and eastern Europe and Latin America [132]. A major problem of the screening programs is the lack of referral compliance, which was strongly detected, as mentioned, in the Kerala program and in the Cuban program [133]. Indisputably, this fact may lead to underreporting of cancer and decreased efficiency of screening programs [134]. The essential reasons for low adherence to definitive diagnostic tests in screening programs are, among others, distance to referral centres, difficulty of transportation, poverty of patients, lack of patient awareness of the importance of diagnosis, and fear of cancer diagnosis and the pain and discomfort that diagnostic tests and treatment will entail [109]. Another major problem that significantly affects the effectiveness of screening programs is the apparent inability of many dentists or family practitioners—due to lack of knowledge, lack of training, or lack of interest—to recognize the warning signs and symptoms that may be indicative of incipient carcinoma [108,135], and the great variability in the level of training of examiners, which has been shown to be very different between specialists in oral Medicine and oral pathology versus general practitioners and general dentists [110,114,133,136]. Likewise, the lack of knowledge on the part of the examiners of the local customs and habits in the areas where the program will be carried out has also been identified as a conditioning factor for the success of a screening program, as this will make it difficult to identify and access the high-risk population [136]. Furthermore, the fact that most screening programs are not randomized controlled trials should be identified as a problem, making it possible to randomize between groups and compare them. The difficulty of conducting randomized controlled trials can be attributed to the length of follow-up required, the reproducibility of the screening test by health care workers, and its high cost [108,135]. Finally, a situation that conditions the low performance of oral cancer screening programs is the level of resources and means of the countries. Countries with a high incidence of oral cancer and a low level of resources also tend to have a low number of primary care physicians and dentists, which makes it necessary to employ health care workers, with lower levels of training than family physicians and dentists, to act as evaluators in screening programs [101]. Although some primary level studies have indicated that these workers can detect oral cancer with similar accuracy to dentists [137], in the opinion of the authors of the present paper, based on their own experience and because of the subtlety of the symptomatology and clinic of incipient cancer, this fact must also constitute a problem in addition to other difficulties that will arise in poor countries and that have been previously mentioned. For Heller et al. [138], this problem could be alleviated by selecting community-integrated primary care workers, providing them with detailed and continuous training and supervision, and authorizing them to prescribe drugs and provide autonomous care. For some authors, these programs present encouraging results [139,140].

All these problems have led important international organizations, such as the UK National Screening Committee or the USPSTF (United States Preventive Services Task Force), not to recommend generalized oral cancer screening in asymptomatic adults. The exception to this is opportunistic screening, with the American Dental Association advising physicians and dentists to perform oral cavity examinations on all patients presenting to their practices [101].

## 5. Final Conclusions and Future Perspectives

This scoping review of systematic reviews on the current state of knowledge regarding delayed diagnosis in oral cancer shows that improving this crucial aspect, with extraordinary repercussions on prognosis, is a major challenge, with poor expectations of being overcome soon. The reasons why an unacceptable percentage of oral cancers are diagnosed late, and therefore in advanced stages and with a poor prognosis, concern each of the events and actors involved in the diagnosis and are often very difficult to correct or even impossible to overcome. The low social and cultural status of the patients conditions an inadequate interpretation of the initial symptoms and signs of oral cancer, and we must sadly recognize that the correction of this aspect in a generalized way constitutes an unattainable utopia. Similarly, the panic that many patients experience when faced with the possibility of having cancer, and which probably transcends their cultural and social status, is a condition of the patient’s own personality that is difficult to change. The reasons concerning health care providers, which condition a delay in the diagnosis of oral cancer, are also difficult to correct in a generalized and rapid manner. The unacceptable lack of knowledge of the initial symptomatology of oral cancer on the part of many primary care physicians and dentists, in the opinion of the authors of this paper, depends essentially on their apathy and lack of commitment to the early diagnosis of this disease. Correcting this unethical attitude is probably also utopian. Moreover, contributing to this, is the fact that the low prevalence of oral cancer in many countries of the world does not favour adequate training of health care providers in this field. The delay in diagnosis due to the overcrowding of public health services, which also causes a delay in the diagnosis and treatment of the disease, is very difficult to correct—for reasons of health policy organization, economics, and the different conception in the organization of the health strategies of the governments that manage these areas in a given country. Finally, the fight against late diagnosis of oral cancer is also hampered by the lack of effectiveness of generalized population screening programs for this disease.

Late diagnosis of oral cancer is probably the main reason why the prognosis of this disease—essentially referring to mortality—has not changed substantially in the last 50 years, despite advances in treatment. The clinicopathological parameters that most powerfully affect prognosis are those that determine the tumour stage at the time of diagnosis, essentially the size and involvement of the neck nodes by the tumour. Large tumours or tumours that already affect lymph nodes at the time of diagnosis have most likely reached this stage for one or more of the following reasons: they have not been detected in time by the patient and/or by the health professional or have been delayed in their treatment due to deficient functioning of the health services. The high mortality that accompanies this situation indicates that the treatment of patients in these stages of cancer is in many cases ineffective; therefore, if we are not able to diagnose oral cancer at earlier stages, the prognosis of the disease, as it is occurring, will foreseeably not improve. What to do then in the face of this bleak outlook? In the opinion of the authors of this paper, there is a need for greater awareness on the part of academic authorities in the field of health—essentially professors in medicine and dentistry—of the need for a more intense and proactive emphasis on oral cancer education and the importance of early diagnosis, which could perhaps involve modifying the curricula of health degrees to give more relevance to this disease; this attitude should also be extended to a greater weight of these aspects in student evaluations. Likewise, government health authorities and professional associations and organizations should implement information programs to increase public awareness of the warning signs of oral cancer and to make patients aware of the importance of regular dental check-ups that include an examination of the oral mucosa. These programs should be repeated periodically. Likewise, the health authorities should improve the functioning of the public health services to alleviate the excessive workload they are subjected to and to improve the time that the different phases of the diagnosis and initiation of treatment of oral cancer must necessarily take. Finally, the States through their different governments, should facilitate and promote the demand for legal responsibilities in those cases in which there is a flagrant delay in the diagnosis of oral cancer.

## Figures and Tables

**Table 1:** Study sample characteristics of systematic reviews and meta-analyses included in this scoping review.

Total Sample	12 Studies
Date of publication	
Range Min (year)	2006
Range Max (year)	2022
Study design	
systematic review	7
systematic review and meta-analysis	5
Study population	
OSCC	10
HNSCC *	2
Primary-level studies included in systematic reviews	
Range min (*n*)	8
Range max (*n*)	63

* Stratified results were reported for oral cancer. Abbreviations: OSCC, oral squamous cell carcinoma; HNSCC, head and neck squamous cell carcinoma.

**Table 2:** Systematic reviews and meta-analyses included in this scoping review, focused on the implications of diagnostic delay in oral cancer (*n* = 12).

Study	Year	Population	Design	Objective(s)	Key Result(s)
Lima et al. [45]	2022	OSCC	SR	To systematically review the causes of the delayed diagnosis of oral cancer mainly in the elderly, in developed and developing countries	Thirteen primary-level studies met the eligibility criteria. All studies included reported causes of delayed diagnosis of oral cancer related to the patient and five also reported causes related to health professionals. The lack of knowledge on oral cancer was pointed out as the main cause of delayed diagnosis. The quality of the evidence was classified as very low for the outcome delayed diagnosis of oral cancer, critically assessed using GRADE system.
Lauritzen et al. [46]	2021	OSCC	SR	To systematically review the literature on the impact of delay in diagnosis and treatment of oral cavity cancer.	Sixteen primary-level studies met the eligibility criteria. Eleven studies examined delay in diagnosis, while five reported a delay in treatment. Eight studies, examining the delay in diagnosis, analysed the impact on prognosis, showing controversial results (three studies found a significant association between patient delay and advanced stage at diagnosis, whereas three others did not). Studies reporting on professional delay and total diagnostic delay, generally, did not find a significant association with advanced cancer at diagnosis. Time to treatment, defined as time from diagnosis to treatment, was also found significantly associated with poor survival in three studies. The quality of the evidence was not assessed or reported.
Walsh et al. [44]	2021a	OSCC	SR + MTA	To estimate the diagnostic accuracy of diagnostic tests for the detection of oral cancer that may provide more timely results, in people presenting with clinically evident suspicious and innocuous lesions.	Sixty-three primary-level studies met the eligibility criteria. None of the adjunctive tests investigated (vital staining, oral cytology, light-based detection, or oral spectroscopy) can be recommended as a substitute for the currently used standard of surgical biopsy and histological evaluation. Most studies reported a minimal time delay between the index test and the reference standard. The quality of the evidence was classified as low or very low for all the outcomes investigated, critically assessed using GRADE system, except for the adjunctive test oral cytology, which obtained a moderate certainty of evidence for the reported pooled sensitivity and specificity.
Walsh et al. [43]	2021b	OSCC	SR + MTA	To estimate the diagnostic accuracy conventional oral examination, vital rinsing, light-based detection, mouth self-examination, remote screening, and biomarkers, used singly or in combination for the detection of oral cancer in apparently healthy adults.	Eighteen primary-level studies met the eligibility criteria. The test accuracy of conventional oral examination may depend on disease prevalence and showed a variable degree of sensitivity (range = 0.50–0.99), with a consistently high specificity (>0.80). Furthermore, there was insufficient evidence to integrate mouth self-examination as part of an organized screening program. In summary, current knowledge does not support the use of screening programmes for oral cavity cancer in the general population. The quality of the evidence was classified as low or very low for most of the outcomes investigated, critically assessed using GRADE system.
Grafton-Clarke et al. [47]	2019	OSCC	SR	To systematically review the knowledge about delays in the diagnosis of symptomatic OSCC in primary care.	Sixteen primary-level studies met the eligibility criteria. In the UK, more than 55% of patients with OSCC were referred by their general practitioner (GP), and 44% by their dentist. Rates of prescribing between dentists and GPs were similar, and both had approximately similar delays in referral. On average, patients had two to three consultations before referral. Less than 50% of studies described the primary care aspect of referral in detail. There was no information on inter-GP–dentist referrals. The quality of the evidence was not assessed or reported.
Varela-Centelles et al. [48]	2017	OSCC	SR	To examine the relative length of the patient and primary care intervals in symptomatic oral cancer.	Twenty-two primary-level studies met the eligibility criteria. The weighted average of patient interval was 80.3 days. Primary care interval was five times shorter (*n* = 15.8 days). The diagnostic interval was shorter (*n* = 47.9 days) when compared with the patient interval during symptomatic period. The quality of the evidence was classified as low for the outcomes evaluated, critically assessed using GRADE system.
Varela-Centelles et al. [30]	2017	OSCC	SR	To identify key points and time intervals in the patient pathway to the diagnosis of oral cancer, from the detection of a bodily change to the start of treatment.	Twenty-eight primary-level studies met the eligibility criteria. These studies generally showed poor methodological quality in terms of questionnaire validation, acknowledgement of biases influencing time-point measurements, and strategies for verification of patient self-reported data. A considerable degree of heterogeneity was also highlighted by the authors. The systematic review findings allowed the definition of key points and time intervals within the Aarhus framework that may better suit the features of the diagnostic process for oral cancer, singularly to assess the impact of waiting time to diagnosis. Although the quality of the evidence was not formally evaluated or reported by the authors, the reported of high risk of bias and the presence of inconsistencies across primary level-studies potentially allows to accept the assumption of a very low quality of evidence, according to GRADE system.
Seoane et al. [12]	2016	OSCC	SR + MTA	To examine the time intervals considered in the studies about diagnostic delay in symptomatic oral cancer and its association to specific outcome measures (survival and TNM classification).	Ten primary-level studies met the eligibility criteria. Regarding referral delay, it was reported a risk increase in mortality of 2.48 (range = 1.39–4.42). The larger the diagnostic delay, the more advanced the stage at diagnosis. High quality studies revealed a higher risk increase than low quality studies (OR = 2.44; 95% CI = 1.36 to 4.36 vs OR = 1.53; 95% CI = 1.26 to 1.86). The quality of the evidence was not assessed or reported.
Seoane et al. [11]	2012	HNSCC	SR + MTA	To address the contradictory information on the role of delay in diagnosis on head and neck cancer survival.	Ten primary-level studies met the eligibility criteria, four of them showing stratified results for oral cancer. Diagnostic delay was not significantly associated with an increased mortality in oral cancer (RR = 1.27; 95% CI = 0.81 to 1.98), according to the authors, this was mainly because two of the studies (50%) restricted their analysis to carcinomas of the tongue. The quality of the evidence was not assessed or reported.
Goy et al. [41]	2009	HNSCC	SR	To examine the evidence for an association between patient and/or provider-related diagnostic delay and late stage at diagnosis in head and neck cancers.	Twenty-seven primary-level studies met the eligibility criteria, 15 of them showing stratified results for oral cancer. The association between diagnostic delay and clinical stage at diagnosis varied in direction and magnitude of the effects, with an inconsistent positive association in oral cancer. The quality of the evidence was not assessed or reported.
Gómez et al. [10]	2009	OSCC	SR + MTA	To systematically review the relationship between total diagnostic delay and advanced clinical stage.	Nine primary-level studies met the eligibility criteria. Diagnostic delay was significantly associated with an advanced clinical stage in oral cancer (RR = 1.47; 95% CI = 1.09 to 1.99). The magnitude of association was higher when meta-analysis was stratified by oral location with a diagnostic delay higher than 1 month (OR = 1.69, 95% CI = 1.26 to 2.77). The quality of the evidence was not assessed or reported.
Scott et al. [42]	2006	OSCC		To systematically review the existing knowledge of factors that influence patient delay in oral cancer.	Eight primary-level studies met the eligibility criteria. The duration of patient delay was generally not associated with clinical factors, tumour parameters, sociodemographic variables, and/or patient health-related behaviours. Patient delay is a problem in oral cancer, but the reasons for such delays are poorly understood and under-researched. The quality of the evidence was not assessed or reported.

Abbreviations: OSCC, oral squamous cell carcinoma; HNSCC, head and neck squamous cell carcinoma; SR, systematic review; MTA, meta-analysis; GRADE, Grading of Recommendations Assessment, Development and Evaluation system; OR, odds ratio; RR, relative risk; CI, confidence intervals.

**Table 3:** Malignant transformation of potentially malignant oral disorders reported in the systematic reviews and meta-analyses published in the special issue organized by the World Health Organization Collaborating Centre for Oral Cancer.

Potentially Malignant Oral Disorders	Sample Size (Primary-Level Studies)	Number of Patients	Malignant transformation *	WHO Collaborating Centre for Oral Cancer Special Issue
Oral leukoplakia	*n* = 24 **	16,192	PP = 9.8% (95% CI: 7.9–11.7)	Aguirre-Urízar et al., 2021
Oral Lichen Planus	*n* = 10 ***	3206	PP = 2.28% (95% CI = 1.49–3.20)	González-Moles et al., 2020
Oral Lichenoid Lesions	*n* = 3	197	PP = 2.11% (95% CI = 0.01–6.33)	González-Moles et al., 2020
Proliferative Verrucous Leukoplakia	*n* = 17	474	PP = 43.87% (95% CI = 31.93–56.13)	Ramos-García et al., 2021
Oral Submucous Fibrosis	*n* = 9	6337	PP = 4.2% (95% CI: 2.7%–5.6%)	Kujan et al., 2020

* This table only integrates those OPDMs for which there is scientific evidence of their malignant transformation poroportions studied through meta-analyses and published in the WHO Collaborating Centre for Oral Cancer special issue. ** Published in the last 5 years. *** Based on 10 highest quality studies selected out of 89 publications. Abbreviations: WHO, World Health Organization; PP, pooled proportions; CI, confidence intervals.

## Data Availability

Data sharing is not applicable to this article as no datasets were generated or analysed during the current study.

## Appendix A. Search Strategy

### Appendix A.1. MEDLINE/PubMed (n = 36)

(“mouth”[MeSH] OR “mouth”[All Fields] OR “oral”[All Fields]) AND (“carcinoma, squamous cell”[MeSH] OR (“carcinoma”[All Fields] AND “squamous”[All Fields] AND “cell”[All Fields]) OR “squamous cell carcinoma”[All Fields] OR “Neoplasms”[Mesh] OR neoplas*[All Fields] OR “cancer”[All Fields]) AND (“Delayed Diagnosis”[Mesh] OR (“delay”[All Fields] AND diagnos*[All Fields]) OR “time interval”[All Fields]) AND (“Meta-Analysis”[pt] OR “meta-analysis”[tiab] OR “Systematic Review”[pt] OR “systematic review”[tiab]).

### Appendix A.2. Embase (n = 86)

(‘mouth’/exp OR ‘mouth’ OR ‘oral’) AND (‘squamous cell carcinoma’/exp OR ‘carcinoma’ OR ‘malignant neoplasm’/exp OR ‘neoplas*’ OR ‘cancer’) AND ((‘delay’ AND ‘diagnos*’) OR ‘time interval’) AND (‘systematic review’:ti,ab OR [systematic review]/lim OR ‘meta-analysis’:ti,ab OR [meta analysis]/lim).

### Appendix A.3. Cochrane Library (n = 8)

((“mouth” OR “oral”) AND (“squamous cell carcinoma”)):ti,ab,kw.

### Appendix A.4. DARE (n = 18)

((“mouth” OR “oral”) AND (“squamous cell carcinoma”)):Any field.
